# TGF-β3 promotes trophoblast development in sheep embryos via ACSS2-dependent permissive lipid metabolism^†^

**DOI:** 10.1093/biolre/ioaf220

**Published:** 2025-10-01

**Authors:** Francesca Boffa, Marika Domenicone, Margherita Moncada, Martina Lo Sterzo, Luca Palazzese, Aurora Scudieri, Emanuele Capra, Barbari Lazzari, Luca Valbonetti, Luisa Gioia, Ramiro Alberio, Domenico Iuso, Pasqualino Loi, Marta Czernik

**Affiliations:** Department of Veterinary Medicine, University of Teramo, Teramo, Italy; Department of Veterinary Medicine, University of Teramo, Teramo, Italy; Department of Veterinary Medicine, University of Teramo, Teramo, Italy; Department of Veterinary Medicine, University of Teramo, Teramo, Italy; Department of Veterinary Medicine, University of Teramo, Teramo, Italy; Department of Veterinary Medicine, University of Teramo, Teramo, Italy; CNR-IBBA, Institute of Agricultural Biology and Biotechnology, National Research Council, Via Einstein, Lodi, Italy; CNR-IBBA, Institute of Agricultural Biology and Biotechnology, National Research Council, Via Einstein, Lodi, Italy; Department of Bioscience and Technology for Food, Agriculture and Environment, University of Teramo, Teramo, Italy; Department of Bioscience and Technology for Food, Agriculture and Environment, University of Teramo, Teramo, Italy; School of Biosciences, University of Nottingham, Sutton Bonington Campus, Nottingham, UK; Department of Veterinary Medicine, University of Teramo, Teramo, Italy; Department of Veterinary Medicine, University of Teramo, Teramo, Italy; Department of Veterinary Medicine, University of Teramo, Teramo, Italy

**Keywords:** blastocyst, in vitro, ovine, tgf-β3, trophoblast

## Abstract

Transforming growth factor-beta (TGF-β) supports the in vitro maintenance of embryonic and trophoblast stem cells. Here, we demonstrated that, in a sheep embryo model, the transition from morula to blastocyst is positively regulated by TGF-β3, primarily through its promotion of trophoblast development. Our results indicate that morulae treated with TGF-β3 develop at a higher rate into blastocysts, characterized by an expanded trophoblast layer marked by CDX-2 expression. In blastocysts, TGF-β3 mediates transcriptional activation of genes involved in cell adhesion and lipid metabolism pathways, leading to remarkable in vitro outgrowth expansion and a substantial increase in trophoblast lipid droplet content. Functional analysis reveals that the positive effects of TGF-β3 are mitigated by inhibition of Acetyl-CoA Synthetase Short-Chain Family Member 2 (ACSS2), a key enzyme upregulated by TGF-β3 and a promoter of de novo lipogenesis. These findings suggest that TGF-β3 modulates lipid metabolism during blastocyst formation and may play a potential role in regulating implantation and placental development.

## Graphical Abstract

**Graphical Abstract:**
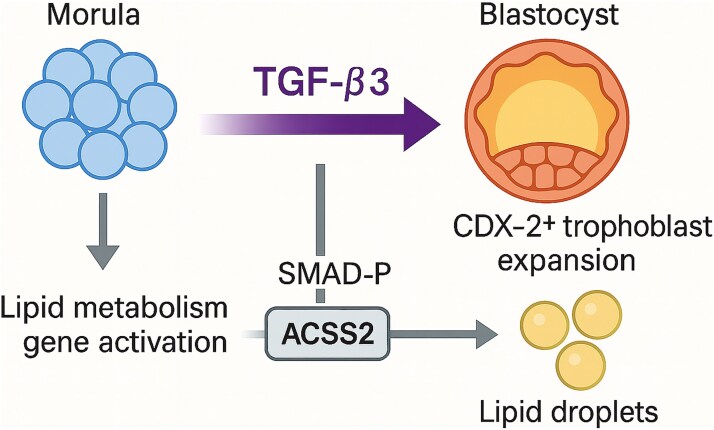

## Introduction

Assisted Reproduction Techniques (ARTs) are widely used in human infertility clinics, the animal industry, and fundamental research. In vitro-produced farm animal embryos serve a pivotal role not only as models for studying human fertility and embryonic development but also in enhancing reproductive efficiency, improving genetic stock, and contributing to the conservation of endangered species [1].

Despite notable advances, current in vitro culture (IVC) systems still fail to achieve similar efficiency as in vivo embryo production [2]. The proportion of embryos that successfully reach the blastocyst stage remains relatively low, often not exceeding 20%–40% in species such as sheep [3], and pigs [4]. A critical bottleneck in in vitro embryo development [5] has been identified during the morula to blastocyst transition, when the inner cell mass and the trophectoderm are established. Despite the significance of this developmental step, its molecular mechanisms remain poorly understood. The transcriptome profile of blastocysts revealing distinct gene expression patterns depending whether they are produced in vivo or in vitro [6]. Furthermore, it was recently reported that metabolomic and epigenetic dysfunctions lead to the arrest of in vitro fertilized embryos [7]. In this study, we investigated the impact of the TGF-β in the morula-to-blastocyst transition in vitro [8]. Previous studies reported TGF-β factors employed as key supplement in culture media for embryonic [9] and trophoblast stem cells [10]. The transforming growth factor-beta (TGF-β) family is defined as a regulator of metabolism, particularly to enhance glucose uptake [11, 12] and as cytokine playing a crucial role in cell differentiation and maintenance of pluripotency [13–15]. TGF-β is characterized by different isoforms, encoded by distinct genes—TGF-β1, TGF-β2, and TGF-β3—each with a molecular weight of approximately 25 kDa and sharing between 71–79% sequence identity [16]. Despite their genetic differences, these isoforms exhibit a highly similar three-dimensional structure, making them nearly indistinguishable in most cell-based reporter gene and growth inhibition assays. Building on this evidence, we hypothesized the TGF-β supports blastocyst formation, potentially leading to more efficient in vitro blastocyst development resembling their in vivo counterparts. Our findings identify a role of TGF-β3 in enhancing in vitro blastocyst development by promoting trophoblast differentiation and adhesion through ACSS2-dependent lipid metabolism. These results suggest that TGF-β3 holds significant potential for improving outcomes in in vitro embryo production offering a promising avenue for enhancing embryo quality and implantation success in both animal and human applications.

## Results

### TGF-β3 enhances in vitro blastocyst development.

**Figure 1:**
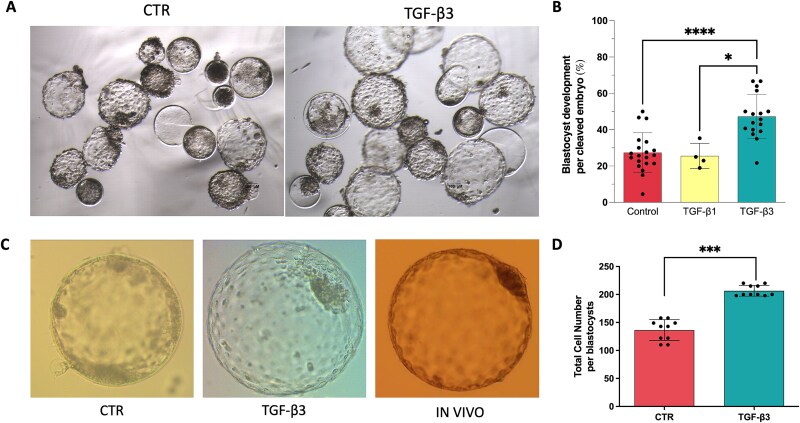
Effect of the treatment with TGF-β1 and TGF-β3 on in vitro embryo development (A) Representative brightfield images of D7 embryos from the control group (CTR) and embryos cultured in presence of TGF-β3, (B) Blastocysts rate (%) on 2-cell stage embryos. ^*^ indicate significant differences (Anova Test: Control vs. TGF-β3 *P* < 0.0001; TGF-β1 vs. TGF-β3 *P* = 0.0207), (C) Representative images of embryos from CTR, TGF-β3, and in vivo recovery groups at equivalent developmental stages. The in vivo blastocyst image is included solely for morphological comparison; no quantification was performed, (D) Graph presenting the Total Cells Number of cells per blastocysts in CTR and TGF- β3 groups (*n* = 10 per group). ^***^ indicate significant differences (Unpaired t test; *P* < 0.001) Bars represent mean ± s.d.

Our first objective was to determine the effects of TGF-β1 and TGF-β3 (0.5 ng/ml) on embryo quality after in vitro fertilization. Embryos were treated from day 4 (morula stage) to day 7 of embryo culture. Although TGF-β1 did not enhance the blastocysts development (Table 1, Figure 1B), supplementation with TGF-β3 led to significant increase of blastocysts rates (45.8% ± 12.5 vs. 28.6% ± 11.8) compared to CTR (Chi-square; *P <* 0.0001) (Table 1, Figure 1B).

Furthermore, blastocysts treated with TGF-β3 exhibited significantly higher cell count (CTR: 136.4 ± 8.86 vs TGF-β3: 206.6 ± 4.53; means ± s.d.; P < 0.001; Figure 1D) and demonstrated improved morphological quality, as evidenced by a higher percentage of hatched embryos on day 7 (Table 1).

**Table 1:** Effect of TGF-β supplementation on embryo development. CTR (*n* = 20), TGF-β1 (*n* = 4), TGF-β3 (*n* = 17). The significant difference is related to the same column (ANOVA test; ^****^ indicate a *P* < 0.0001)

Group	*2cells/Oocytes (%)*	*Blastocysts/2cells (%)*	*Hatched at D7/Total blastocysts (%)*
*CTR*	379/685 (55.3% ± 17.2)	100/379 (28.6% ± 11.8)	16/100 (16% ± 0.8)
*TGF-β1*	205/376 (56.7% ± 15.3)	51/205 (25.6% ± 15.3)	9/51 (17.6% ± 2.4)
*TGF-β3*	395/685 (57.6% ± 15.2)	181/395 (45.8% ± 12.5)	79/181 (43.6% ± 4.6)^****^

### TGF-β3 treatment promotes trophoblast expansion in vitro.

**Figure 2:**
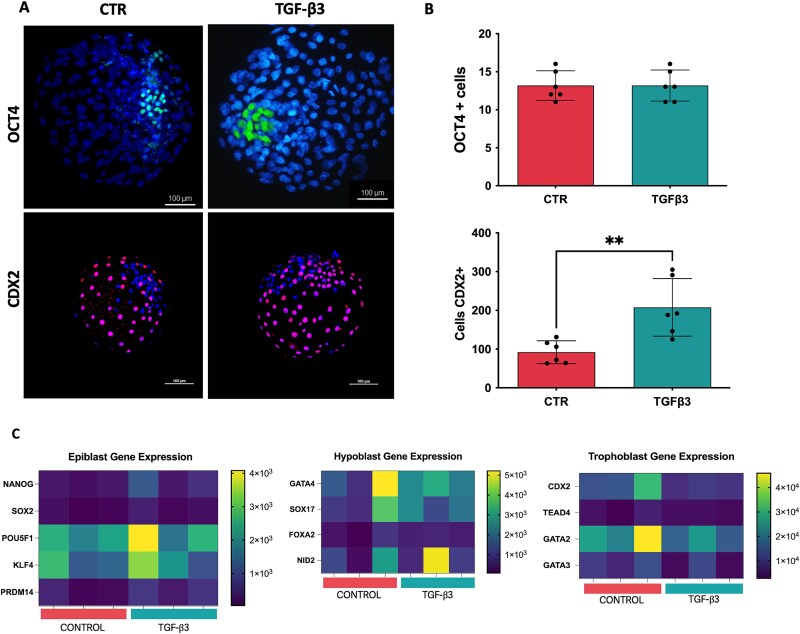
Epiblast and Trophoblast development in D7 embryos (A) Representative immunostaining of OCT4, a hallmark gene of the Inner Cell Mass (ICM), and CDX2, a marker indicative of Trophoblast cells, in control (CTR) and TGF-β3-treated blastocysts at day 7. (B) Quantification of the number of cells positive for (OCT4 CTR OCT4+: 13.6 ± 2 vs. TGF-β3: 13.2 ± 2) and CDX2 (CTR CDX2+: 95.3 ± 33.8 vs. TGF-β3: 204.5 ± 78.3); *n* = 6 for each group (Unpaired t test; *P* ≤ 0.01). (C) Heatmap of expression levels of selected lineage-specific genes (log2-normalized gene counts)(*n* = 3 replicates of single blastocysts for each group).

Immunofluorescence analysis for CDX2 and OCT4 revealed a significant increase in the number of CDX2-positive cells, which marks the trophectoderm layer, in the TGF-β3 treated blastocysts compared to the control group (Figure 2A and B). In contrast, the number of OCT4-positive cells, marking the epiblast, remained unchanged (CTR: 13.6 ± 2 vs TGF-β3: 13.2 ± 2; means ± s.d.; Figure 2A and B). However, despite the observed increase in trophectoderm cell numbers, transcriptomic analysis did not reveal any significant upregulation of lineage-specific gene expression associated with the three major lineages of the embryo at this stage of development (Figure 2C). This suggests that while TGF-β3 promotes trophectoderm proliferation, it does not appear to influence the molecular differentiation pathways governing lineage specification in blastocysts (Figure 2B and C) [17, 18].

### Transcriptome analysis of the TGF-β3 treated blastocysts

**Figure 3:**
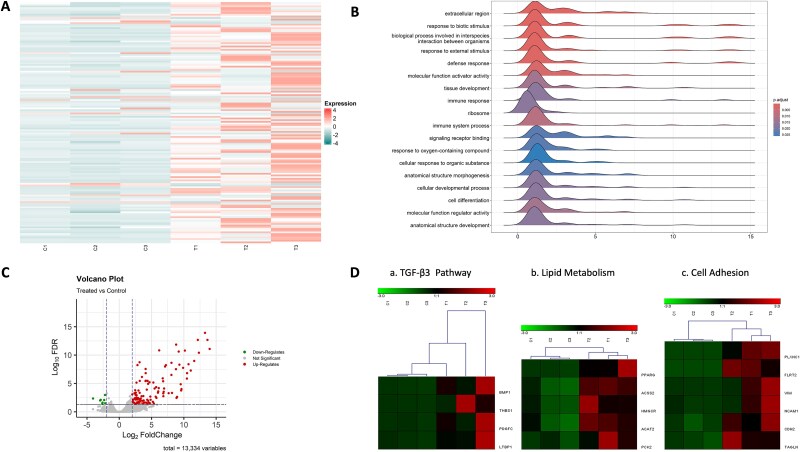
Transcriptome analysis: (A) Heatmap displaying 144 differentially expressed genes (DEGs) for each group (*n* = 3). (B) Ridgeline plots depicting significantly enriched KEGG pathway gene sets (padj < 0.01) among DEGs in the TGF-β3-treated group. (C) Volcano plot illustrating significant DEGs with Log2 fold change and *P* < 0.01. (D) Classification of key gene groups within TGF-β3-treated DEGs, associated with: (a) TGF-β signaling, (b) lipid metabolism, and (c) cell adhesion.

A total of 638 million paired-end reads were obtained from all samples (three CTR, three TGF-β3), averaging 106.4 million ±33.6 million reads per sample. After quality filtering, an average of 91.7% of high-quality paired reads were successfully mapped to the sheep reference genome ARS-UI_Ramb_v2.0. Generalized PCA, considering a total of 21 303 unique genes, revealed that gene expression varied after TGF-β3 treatment, with a complete separation between CTR and TGF-β3 samples along PC2, which explained approximately 23.9% of the variance. A total of 144 differentially expressed genes (DEGs) were identified in TGF-β3-treated blastocysts, with the majority being upregulated (*n* = 129) and only 15 downregulated compared to CTR (Figure 3A). To gain insights into the biological response of blastocysts following treatment, a functional enrichment analysis was performed on the DEGs. Notably, the expression of BMP1 [19], THBS1 (Thrombospondin-1) [20], PDGF-C [21], and LTBP1 (Latent Transforming Growth Factor Beta Binding Protein 1) [22]—all intricately linked to or regulated by the TGF-β signaling pathway—strongly indicates that the TGF-β pathway was effectively activated in our system (Figure 3B).

Moreover, when focusing on the pluripotency gene network between the groups, we observed no significant differences in the expression of KLF4, MYC, NANOG, OCT4 (POU5F1), and SOX2 between the blastocysts from both groups (Figure 2C). This suggests that while TGF-β3 promotes the embryo formation and leads to an increase in morula development (approximately 15%–20% more morulae), it maintains the same level of pluripotency and guides their development into blastocysts (Figure 1C) [23].

Two groups of overexpressed genes cover functional expression clusters that caught our attention. The first group formed by PLXNC1 (Plexin C1) [24], FLRT2 (Fibronectin Leucine Rich Transmembrane Protein 2) [25], VIM (Vimentin) [26], NCAM1 (Neural Cell Adhesion Molecule 1) [27], CDH2 (Cadherin 2) [28, 29], TAGLN (Transgelin) [30] related to blastocyst and cells adhesion, represents the most upregulated genes in TGF-β3 blastocysts [31]. The second group, including PPARG (Peroxisome Proliferator-Activated Receptor Gamma), ALDH1L2 (Aldehyde Dehydrogenase 1 Family Member L2), ACSS2 (Acyl-CoA Synthetase Short Chain Family Member 2), HMGCR (3-Hydroxy-3-Methylglutaryl-CoA Reductase), ACAT2 (Acetyl-CoA Acetyltransferase 2), e PCK2 (Phosphoenolpyruvate Carboxykinase 2, Mitochondrial) suggested that the lipid metabolism pathways are significantly increased in TGF-β3 blastocysts [32].

### Effect of TGF-β3 treatment on blastocyst outgrowth

**Figure 4:**
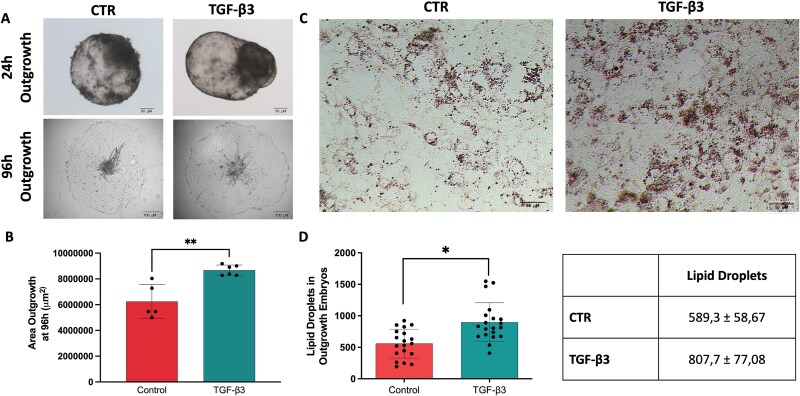
Outgrowth expansion and Lipid Metabolism involvement (A) Representative images of blastocyst outgrowth at 24 h post-attachment to a gelatin-coated plate and at 96 h. (B) The outgrowth area at 96 h was measured in μm^2^ for blastocysts cultured in outgrowth medium. (CTR mean: 6.4 ×106 μm ± 3.1 ×106 vs TGF-β3 mean: 8.8 × 106 μm ± 0.6 ×106); Data were analyzed using Fiji software and are presented as the mean ± standard deviation (s.d.). (^**^) indicate significant differences (Kolmogorov–Smirnov test; *P* ≤ 0.009). Every dot represents the replicate involved in the analysis. (C) Lipid deposition in outgrowth tissues was visualized using Oil Red O staining at days 13 and 14. (D) Lipid droplets were quantified using Fiji software (ImageJ), with results shown as the mean ± s.d. (^*^) indicates significant differences (Unpaired t test; *P* ≤ 0.03).

**Figure 5:**
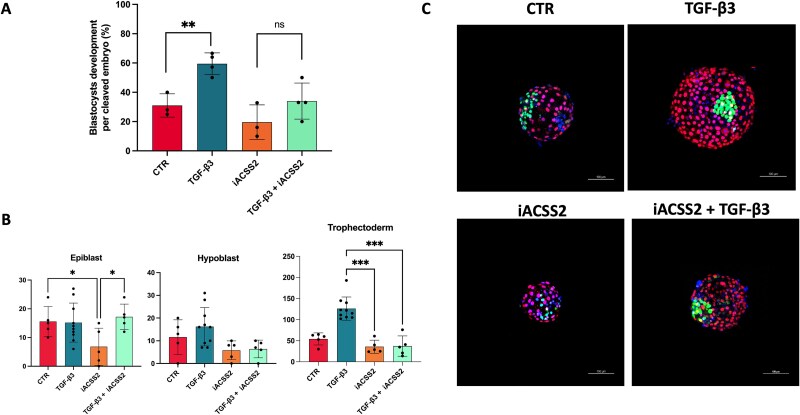
Impact of lipogenesis inhibition on TGF-β3 induced blastocysts development (A) Blastocyst rate (%) from 2-cell stage embryos. ^*^ indicates significant differences (ANOVA test; Control vs. TGF-β3, *P* = 0.0268; TGF-β3 vs. iACSS2, *P* = 0.0051; TGF-β3 vs. TGF-β3 + iACSS2, *P* = 0.0344). Bars represent mean ± s.d. Each dot represents an independent replicate. Blastocyst development rates were: Control 31%, TGF-β3 59.4%, iACSS2 19%, TGF-β3 + iACSS2 34% (B) Quantification of the number of cells positive for SOX2, CDX2, and unmarked cells, identified as hypoblast cells. Significant differences were determined using ANOVA (CDX2-positive cells: TGF-β3 vs. iACSS2, *P* = 0.0061; TGF-β3 vs. TGF-β3 + iACSS2, *P* = 0.0073; SOX2-positive cells: Control vs. iACSS2, *P* = 0.0401; iACSS2 vs. TGF-β3 + iACSS2, *P* = 0.0150). Mean cell counts were: SOX2 – Control 15.6 ± 5.2, TGF-β3 14 ± 6.8, iACSS2 6.8 ± 6.4, TGF-β3 + iACSS2 24.3 ± 15.3; CDX2 – Control 54.2 ± 14.2, TGF-β3 96.9 ± 45.3, iACSS2 35.8 ± 15.2, TGF-β3 + iACSS2 38 ± 27.8. Hypoblast cells were calculated by subtracting SOX2+ and CDX2+ cells from the total cell count. Bars represent mean ± s.d. (C) Representative immunostaining of SOX2 (green), CDX2 (red), and nuclear staining with DAPI (blue) in blastocysts at day 7 for the four experimental groups.

In vitro blastocyst outgrowth is a reliable model to predict implantation potential in vivo [33]. Although ruminant embryos typically attach around day 17–20 post-fertilization and undergo a distinct elongation phase prior to implantation, the in vitro outgrowth system intentionally accelerates and simplifies this process by promoting trophoblast adhesion on a culture substrate. Consequently, while it does not fully replicate the elongated conceptus stage, it remains a powerful tool for exploring the early adhesion and invasion properties of trophoblast cells relevant to implantation. Our results showed that TGF-β3-treated blastocysts exhibited significantly higher attachment rates and outgrowth expansion compared to controls (CTR) (5/16 [31.2%] in CTR and 7/14 [50%] in TGF-β3) (Figure 4A and B). On day 4 after the initiation of outgrowth, we measured the growth rate by calculating the area of both CTR and TGF-β3-treated embryos. We determined a significantly larger area in TGF-β3-treated blastocysts compared to controls (Figure 4B). The predominant cells observed in these cultures displayed epithelial-like morphology, characterized by dark cytoplasm and distinct cell boundaries. To confirm transcriptomic analysis indicating that TGF-β3 treatment may affect lipid metabolism, we performed Oil Red O staining. As shown in Figure 5C and D, the TGF-β3 group contained a significantly higher number of lipid droplets compared to the control group, suggesting enhanced lipid metabolic activity (Figure 4C and D).

### TGF-β3 promotes blastocyst development through ACSS2 activity

To investigate whether the improvement observed with TGF-β3 treatment was directly linked to de novo lipogenesis, as suggested by the transcriptome analysis, we inhibited key metabolic pathways at the morula stage. In particular, we used Ac-CoA Synthase Inhibitor 1 (MedChemExpress 508186-14-9) at a concentration of 6 μM to block ACSS2 activity, the enzyme responsible for converting acetate to acetyl-CoA in lipid metabolism, whose expression was upregulated in TGF-β3-treated blastocysts (Figure 3D). The results showed that the positive effects of TGF-β3 on blastocyst development were significantly reduced by the metabolic inhibitors (Figure 5A). This suggests that the enhancement of blastocyst development by TGF-β3 is mediated by ACSS2 activity guiding the lipid metabolism. To further examine epiblast and trophoblast expansion in these embryos, we stained them with SOX2, as a pluripotency and epiblast marker, and CDX2, as a trophoblast marker [34] (Figure 5B and C).

**Figure 6:**
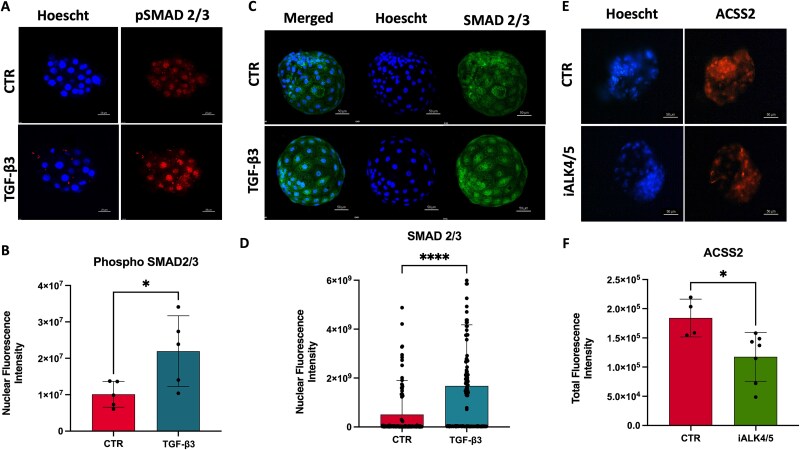
TGF-β3 activates SMAD2/3 phosphorylation and promotes ACSS2 expression in early sheep embryos. (A) Representative image of a morula cultured for 16 h with 0.5 ng/ml TGF-β3, showing nuclear accumulation of phosphorylated SMAD2/3 (p-SMAD2/3) by immunostaining. (B) Quantification of nuclear p-SMAD2/3 intensity per morula (*n* = 5 embryos per group); values represent mean nuclear intensity per embryo. Statistical analysis: unpaired t test (*P* = 0.0500). (C) Representative day 7 blastocysts showing enhanced nuclear localization of p-SMAD2/3. (D) Nuclear p-SMAD2/3 intensity per cell nucleus (*n* = nuclei from three blastocysts per group). Statistical analysis: Kolmogorov–Smirnov test (*P* < 0.0001). (E) Representative blastocyst at day 7 treated with ALK5/ALK4 kinase inhibitor (SM16) at 20 μM, showing reduced ACSS2 signal in both nucleus and cytoplasm, compared to DMSO-treated control. (F) Quantification of total ACSS2 fluorescence intensity per blastocyst (*n* = 4 control, *n* = 6 SM16-treated). Statistical analysis: unpaired t test (*P* = 0.0263). Each dot represents one embryo or nucleus, as indicated.

To validate the link between TGF-β3 signaling and ACSS2 expression, we next inhibited the phosphorylation of SMAD2/3—key downstream effectors of canonical TGF-β signaling [Shen et al., 1998]—using SM16 (ALK5/ALK4 kinase inhibitor) at 20 μM in morula stage. SM16 treatment markedly reduced the nuclear localization of phosphorylated SMAD2/3 in blastocysts, indicating effective pathway inhibition (Figure 6A–D, Supplementary Figure 1). Interestingly, this reduction in p-SMAD2/3 signal was accompanied by a decrease in ACSS2 protein levels (Figure 6E and F), further supporting the hypothesis that TGF-β3 promotes ACSS2 expression via SMAD2/3 activation.

## Discussion

In this study, we show that TGF-β3 enhances sheep blastocyst development in vitro. The TGF-β/Activin/NODAL signalling pathway is critical for pattern formation and differentiation, particularly during the pregastrulation and gastrulation stages of mammalian development [35]. TGF-β is produced in the uterus during embryo development, coinciding with blastocyst development [36]. While TGF-β isoforms are typically similar in most cell-based assays gene expression and growth inhibition, TGF-β2 has been noted as an exception, exhibiting 100–1000 time lower potency than TGF-β1 and TGF-β3 in cell lines lacking TβRIII [37, 38]. Interestingly, TGF-β1 may exert more pronounced effects at later embryonic stages, as inhibiting TGF-β1 receptor kinase (SB431542) prevents hypoblast migration and epiblast proliferation in later-stage ovine embryos [39], suggesting a potentially distinct developmental window for TGF-β1 compared with TGF-β3.

Our pilot screening using TGF-β1 and TGF-β3 showed TGF-β3 significantly improved embryo progression from the morula stage. These blastocysts closely resembled the cell number reported from in vivo-produced ones (160–180). In fact, TGF- β3 treatment increased the number of CDX2-positive TE cells in blastocyst, while the number of OCT4-positive epiblast cells remained unchanged. Blastocysts generated by TGF-β3 treatment exhibited a balanced mRNA expression of key markers for the epiblast (SOX2, OCT4, NANOG, KLF4, PRMD14), trophoblast (CDX2, TEAD4, GATA2, GATA3), and hypoblast (SOX17, GATA4, FOXA2, NID2), as confirmed by bulk RNA-seq analysis, closely mirroring control blastocysts. Furthermore, immunostaining for OCT4 and CDX2, along with gene expression analysis, revealed no significant differences also in the protein levels of these lineage markers. We conclude that TGF-β3 treatment at day 4 of IVC supports the progression of morula to blastocysts by promoting the TE lineage.

Among all the genes highlighted in the blastocysts transcriptome analysis, Cadherin 2 and Vimentin stand out as key regulators of cell–cell adhesion. Cadherin 2 plays an essential role in establishing strong cell–cell adhesion for maintaining the integrity of the developing embryo, facilitating the morphogenetic movements that shape the blastocyst and its implantation [28, 29, 40, 41]. Similarly, Vimentin, an intermediate filament protein, is highly expressed in mesenchymal cells, supporting processes as cell migration, adhesion, and maintenance of cell shape, all of which are vital during embryonic development [26, 42]. The upregulation of other genes associated with blastocyst adhesion, such as PLXNC1 [24], Fibronectin Leucine-Rich Transmembrane Protein 2 [25], NCAM1 [26], and TAGLN [27], along with enhanced TE development, supported the hypothesis that TGF-β3-treated blastocysts may exhibit improvement in adhesion and potentially in implantation success.

To validate this, we performed outgrowth experiments to simulate the in vivo attachment to uterus. The results confirmed that treated blastocysts exhibited an adhesion capacity three times greater than control blastocysts indicating a higher likelihood of successful peri-implantation development.

Furthermore, the analysis of the DEGs in TGF-β3-treated blastocysts revealed higher expression of key genes associated with lipid metabolism such as PPARG, ACSS2, PCK2, and ALDH1L2. Peroxisome Proliferator-Activated Receptor Gamma (PPARG) is reported as a key transcription factor regulating genes involved in lipid biosynthesis [43] and was notably overexpressed during the elongation blastocysts phase of sheep conceptus development [44–46].

Its increase in TGF- β3 trophoblast cells was further emphasized by the notable accumulation of lipid droplets in TGF- β3-treated trophoblast cells [47]. While increased lipid content in in vitro-derived embryos is often associated with reduced cryotolerance [48], it is essential to consider the physiological role of lipid droplets in early embryonic development. The upregulation of genes like PPARG and ACSS2 suggests a regulated metabolic adaptation rather than a sign of cellular stress. Lipid droplets may serve as an energy reservoir, supporting the trophoblast’s metabolic demands during the peri-implantation period. Genetic ablation of PPARG in mice results in embryonic lethality due to placental defects, which can be rescued by a wild-type placenta [49, 50]. Moreover, reducing PPARG activity has been associated with preeclampsia [51], and consistently, in ruminant, the knockout of embryonic PPARG leads to a reduction in the total cell number in D7.5 blastocysts [52]. Despite challenges in achieving the elongation blastocyst stage in ruminants using IVC systems, our findings suggest that PPARG expression with the activation of lipid metabolism is a crucial pathway for blastocyst progression and elongation.

Among the overexpressed genes related to de novo lipogenesis, in combination PPARG, our focus was on ACSS2, which has been reported to be hyperactivated by TGF- β signaling [53]. ACSS2 has previously been described as a principal producer of Acetyl-CoA from acetate during embryo development and in trophoblast stem cells under metabolic stress [34, 54]. Repression of SMAD2/3 phosphorylation—the downstream effectors of canonical TGF-β pathway—using an ALK5/ALK4 kinase inhibitor led to reduced nuclear SMAD2/3 levels in blastocysts, accompanied by a significant decrease in ACSS2 protein expression. These findings suggest that TGF-β3 positively regulates ACSS2 expression via SMAD2/3 activation. In fact, the inhibition of ACSS2 decreased blastocyst development and mitigated the positive effects of TGF-β3 reducing its ability to enhance trophoblast lineage expansion, as shown by a marked depletion in CDX2-positive cell numbers. When de novo lipogenesis support declined, the beneficial outcomes achieved with TGF-β3 treatment were substantially reduced, failing to replicate the developmental progression observed in the untreated controls. Despite progress, the underlying mechanisms regulating these metabolic pathways remain to be fully elucidated.

Our study reveals that TGF-β3 supports in vitro blastocyst formation by promoting ACSS2-dependent lipid metabolism via SMAD2/3 activation, thereby facilitating trophoblast development and contributing to lipid metabolic adaptation in early embryonic development. Although our study is predominantly in vitro, the promising results suggest that TGF-β3 is a valuable candidate for advancing embryo production; consequently, further in vivo experiments—such as assessing pregnancy rates following embryo transfer—are warranted to confirm and extend its potential impact on ARTs as well as on peri- and post-implantation development.

## Material and methods

### Animals and ethics approval

All animal experiments (semen collection) have been approved by the Italian Ministry of Health, upon the presentation of the research description prepared by the ethics committee of the Istituto Zooprofilattico Sperimentale di Teramo (Prot. 944F0.1 del 04/11/2016). The number of the authorization granted by the Italian Ministry of Health is no 200/2017-PR. All methods were performed in accordance with the relevant guidelines and regulations of the Italian Minister of Health.

### Embryo production

#### Oocyte collection and in vitro maturation

Sheep ovaries were ethically sourced from local slaughterhouses and immediately transported to the laboratory within a 1–2 h, maintaining a temperature of 37°C. Oocytes were aseptically extracted using 21 G needles in the presence of H-199 medium (Sigma-Aldrich, M7528), which was supplemented with 10% Bovine Serum Albumin (BSA), Gentamicin solution, and heparin. Oocytes possessing a minimum of 2–3 layers of compact cumulus cells were meticulously selected for in vitro maturation (IVM). Maturation was conducted in bicarbonate-buffered TCM-199 medium (Gibco) containing 2 mM glutamine, 0.3 mM sodium pyruvate, 100 μM cysteamine, 10% fetal bovine serum (FBS) (Gibco), 5 μg/ml follicle-stimulating hormone (FSH), 5 μg/ml luteinizing hormone (LH), and 1 μg/ml estradiol [55]. The IVM process occurred in 4-well culture plates, with each well containing 0.5 ml of IVM medium and approximately 30 oocytes per well, incubated in a humidified atmosphere at 38.5°C with 5% CO2 for 22 h. Following IVM, only carefully selected MII oocytes exhibiting expanded cumulus and normal morphology were employed for in vitro fertilization (IVF) [56].

#### In vitro fertilization and IVC

22 h after IVM, cumulus-oocyte complexes (COCs) displaying cumulus cell expansion were exclusively chosen for IVF. These COCs were gently transferred into a solution containing 300 U/ml of hyaluronidase dissolved in H-199, washed twice in H199, and then placed in 50 μl droplets (with 10 oocytes per drop) of IVF medium (SOF-supplemented with 20% oestrus sheep serum and 16 mM isoproterenol), which were covered with mineral oil. A single straw of frozen semen, containing 100 × 10^6^ spermatozoa, was rapidly thawed in 35–37°C water and subjected to centrifugation in sperm-wash medium (bicarbonate-buffered synthetic oviductal fluid (SOF-) with 0.4% (w:v) fatty-acid-free BSA) at 300 g for 5 min. The supernatant was discarded, and 5 × 10^6^ spermatozoa were added to each drop, followed by incubation in a humidified environment at 38.5°C, 5% CO_2_, and 7% O_2_ [56]. Within the initial 4 h following IVF, meticulous oocyte cleansing was carried out by gentle pipetting in a solution of SOF-enriched with 2% (v:v) basal medium Eagle essential amino acids (EAA), 1% (v:v) minimum essential medium (MEM)-nonessential amino acids (NEAAs) (Gibco), 1 mM glutamine, and 8 mg/ml fatty acid-free BSA (SOF-aa). This process aimed to remove granulosa cells. Subsequently, the oocytes underwent three additional rinses in an IVC medium (BO-IVC Embryo Culture Medium, IVF Bioscience) before being grouped in culture drops of 20 μl, with each group containing five oocytes. On day 7 of culture, the assessment of blastocysts was carried out using a Nikon Eclipse Ti2-U inverted microscope, aided by the Octax EyeWare Imaging Software (version 2.3.0.372).

### Experimental design

On day 4 of culture defined as day 0 from fertilization, embryos at the compact morula stage were divided into two experimental groups. The first group was treated with either recombinant human TGF-β1 PLUS protein (Qk010) or Transforming Growth Factor-β3 human (T5425-2UG) at a concentration of 0.5 ng/mL, which was added to the IVC medium. This concentration was selected based on preliminary pilot experiments and by referencing the half maximal effective concentration (EC50), ensuring that the experimental conditions were optimized for reliable results [57]. A separate group of embryos was cultured in standard IVC medium without TGF-β supplementation, serving as the CTR for all subsequent experiments.

### Treatment of sheep blastocysts with small-molecule inhibitors

The small-molecule inhibitors Ac-CoA Synthase Inhibitor 1 (MedChemExpress 508186-14-9), targeting acetate-dependent acetyl-CoA synthetase 2 (ACSS2), and SM16 (MedChemExpress HY-111482), an ALK5/ALK4 kinase inhibitor, were dissolved in dimethyl sulfoxide (DMSO; Sigma-Aldrich, D2650) to prepare stock solutions at 10 mM (ACSS2 inhibitor) and 20 mM (SM16), respectively. The inhibitors were then diluted to the desired concentration in pre-equilibrated, supplemented IVC medium (BO-IVC Embryo Culture Medium, IVF Bioscience).

### Embryo evaluation and total cell count

Embryos were assessed on day 7 of culture, selecting those that had successfully hatched by this point to ensure the selection of the most advanced and viable embryos. After fixation, the total number of cells of each embryo was determined using DAPI staining to visualize the nuclei. Quantification of the total number of cells was performed using QuPath software version 0.5.1, which allowed for precise cell counting.

### Immunofluorescence

Embryos were fixed in 4% paraformaldehyde for 15 min, washed in PBS with 1% bovine serum albumin (BSA), permeabilized in 1% Triton X-100 in PBS for 15 min at room temperature (RT) and blocked in 5% BSA and 1% Tween20 in PBS for 1 h at RT. Then, they were incubated overnight at 4°C with primary antibodies (Supplementary Table 1). After four washes in 1% BSA in PBS, embryos were incubated in the appropriate secondary Alexa-conjugated antibodies (Supplementary Table 1) and counterstained with DAPI for 1 h at RT, followed by four washes in 1% BSA in PBS. Finally, embryos were mounted and imaged (Nikon Ar1 laser confocal scanning309 microscope (Nikon Eclipse Ti-E) equipped with the NIS- Element 4.40 software).

### Immunostaining for detection of phosphorylated SMAD proteins

All samples were washed using 0.1% Triton in PBS- and fixed using 4% paraformaldehyde in PBS+ for 1 h on ice. The samples were incubated for 5 min each in a dilution series of 25, 50, 75% and pure methanol (Fisher Scientific, Cat #67-56-1) in 0.1% Triton PBS. The dilution series was then reversed to full rehydration in 0.1% Triton PBS-. The samples were then incubated in ice-cold acetone (Fisher Scientific, Cat #67-64-1) for 20 min at −20°C. After acetone treatment, the samples were briefly washed in 0.1% Triton PBS- and permeabilised in 1% Triton-PBS- at RT for 20 min. Application of blocking solution, primary, and secondary antibodies was identical to the description above. Primary antibodies rabbit anti-pSMAD2 were applied at 1:50 dilution. pSMAD proteins were detected using goat-raised, Alexa-647 conjugated secondary antibodies. Argon laser excitation yielded optimal detection of pSMAD immunofluorescence.

### In vitro outgrowth assay

An outgrowth assay was performed on both TGF- β3-treated and CTR blastocysts. Blastocysts were placed on gelatine-coated plates and cultured in outgrowth medium consisting of DMEM supplement with 10% of FBS, 0.1% glutamine, 0.05% NEAAs, 0.05% gentamicin and 1 μL/mL FGF2.

Each blastocyst (one per well) was transferred to the gelatin-coated plates using a mouth capillaries. Since the blastocysts did not attach to the gelatin layer spontaneously, on day 2 of culture, mechanical implantation was simulated using two fine needles to gently secure the blastocysts onto the surface. The blastocyst were then cultured for an additional two days before imaging. The outgrowth area was analyzed by processing the images with ImageJ software to measure the expansion of the blastocyst outgrowth. Embryos were monitored daily, and the medium was refreshed every 2–3 days. Photographic documentation was captured throughout the culture period to track the progression of the outgrowth.

### Oil red O staining for lipid droplet detection

Lipid droplet accumulation in outgrown cells derived from sheep blastocysts was evaluated using Oil Red O staining. Cells cultured in 4-well plate were initially washed with DPBS and then fixed with 4% paraformaldehyde for 30 min. After fixation, the cells were stained with 5 μg/mL Oil Red O solution (Sigma, USA) for 10 min, following the protocol previously described [58]. After staining, the cells were carefully rinsed to remove excess stain and were then examined using light microscopy to visualize the presence and distribution of lipid droplets within the cells. Photographs were taken to document the results for further analysis.

### Transcriptome analysis

#### RNA extraction

RNA was isolated from three biological replicates for each condition (*n* = 3 for control and *n* = 3 for TGF-β3), using the NucleoSpin miRNA kit (Macherey–Nagel) following the protocol in combination with TRIzol lysis (Invitrogen) and recovery of small and large RNA recovery in one fraction. The quality and quantity of RNA were determined by Agilent 2100. The isolated RNAs were stored at −80°C until use.

#### Library preparation

RNA samples (RIN &gt; 6.5) from three replicates (*n* = 3) for each treatment (*n* = 2, control C, Treated with TGF-β3 T) were used for library preparation. RNA-Seq libraries were obtained using the Illumina TruSeq RNA Sample Preparation Version2 kit after polyA enrichment, and libraries enrichment with 22 cycles of PCR, accordingly to what previously reported for sequencing low input translatome samples [59]. Concentration and quality checks of libraries were determined using Agilent 2100 bioanalyzer. Sequencing was performed on Illumina NovaSeq X, 150 cycles paired end.

#### Data analysis

The nf-core/rnaseq version 3.8.1 pipeline (https://nf-co.re/rnaseq) was run for RNA-Seq data analysis. The pipeline integrates the sequence trimming (TrimGalore version 0.6.7) and sequence alignment (STAR version 2.7.10a), [60]. Sequences were aligned to the *Ovis Aries* ARS-UI_Ramb_v2.0 reference genome and alignments to gene regions were quantified using Salmon version 1.5.2 (https://combine-lab.github.io/salmon/). Differential expression between samples were calculated with The EdgeR Bioconductor package version 3.6 (Bioconductor, https://bioconductor.org/packages/release/ bioc/html/edgeR.html) [61]. Principal Component Analysis PCA and hierarchical clustering were performed with Genesis [62].

#### Quantitative PCR qPCR

4 μl of RNA was added of 0.125 μl of random hexamers, 0.5 μl oligodT, 0.5 μl dNTPs and 5 μl RNAse free water and incubated at 65°C for 5 min and placed at 4°C. Mixture was then added of 4 μl RT buffer (5 x), 1 μl of DTT, 1 μl RNase inhibitor, and 1 μl of SuperScript II Reverse Transcriptase (Thermo Fisher, Waltham, MA USA). Reverse transcription was carried out at 25°C for 5 min, 42°C for 1 h and 70°C for 15 min. The transcript level of the different MX Dynamin Like GTPase 1 (MX1), Inhibin Subunit Alpha (INHA) and Bromodomain and WD Repeat Domain Containing 3 (BRWD3) genes and glyceraldehyde- 3-phosphate dehydrogenase (GAPDH), actin beta (ACTB) as a housekeeping gene, were measured using real time-PCR. Primers were designed from specific exon-exon junction to avoid DNA genomic. Real-time PCR was performed using 4 μL of diluted cDNA (1:20 Vol.), 5 μl of the SYBR Green Master Mix (Applied Biosystems, Carlsbad, California, USA) and 0.5 μl of forward and reverse primers (final concentration 900 nM with QuantStudio 6 Flex Real-Time PCR Systems (Applied Biosystems, Carlsbad, California, USA). Gene relative expression between C and T groups were calculated by the PCR R package [63].

### Statistical analysis

Quantitative data are expressed as mean ± SD. If parametric, Unpaired t test was used to compare the averages/means of two conditions, if not-parametric, Kolmogorov–Smirnov test was used to determine if there is a significant difference between the two groups. Developmental rate were compared by chi-square test. Statistical significance was considered when probability values *(P)* were lower than 0.05. Statistical analyses were performed using GraphPad Prism software (Version 10.4.1, CA, USA).

## Supplementary Material

Supplementary_Figure_ioaf220

Supplementary_Table_ioaf220

## Data Availability

RNA-seq data are deposited in NCBI accession BioProject ID: PRJNA1193800. Further information and requests for resources and reagents should be directed to and will be fulfilled by the lead contact.
