# Supplementary material for: TGF-β3 promotes trophoblast development in sheep embryos via ACSS2-dependent permissive lipid metabolism
Source: Biol Reprod. 2025 Oct 1;114(3):773–83. doi: 10.1093/biolre/ioaf220 (PMC13017937; doi:10.1093/biolre/ioaf220)
Supplement: Supplementary_Figure_ioaf220 [file supplementary_figure_ioaf220.docx]

**
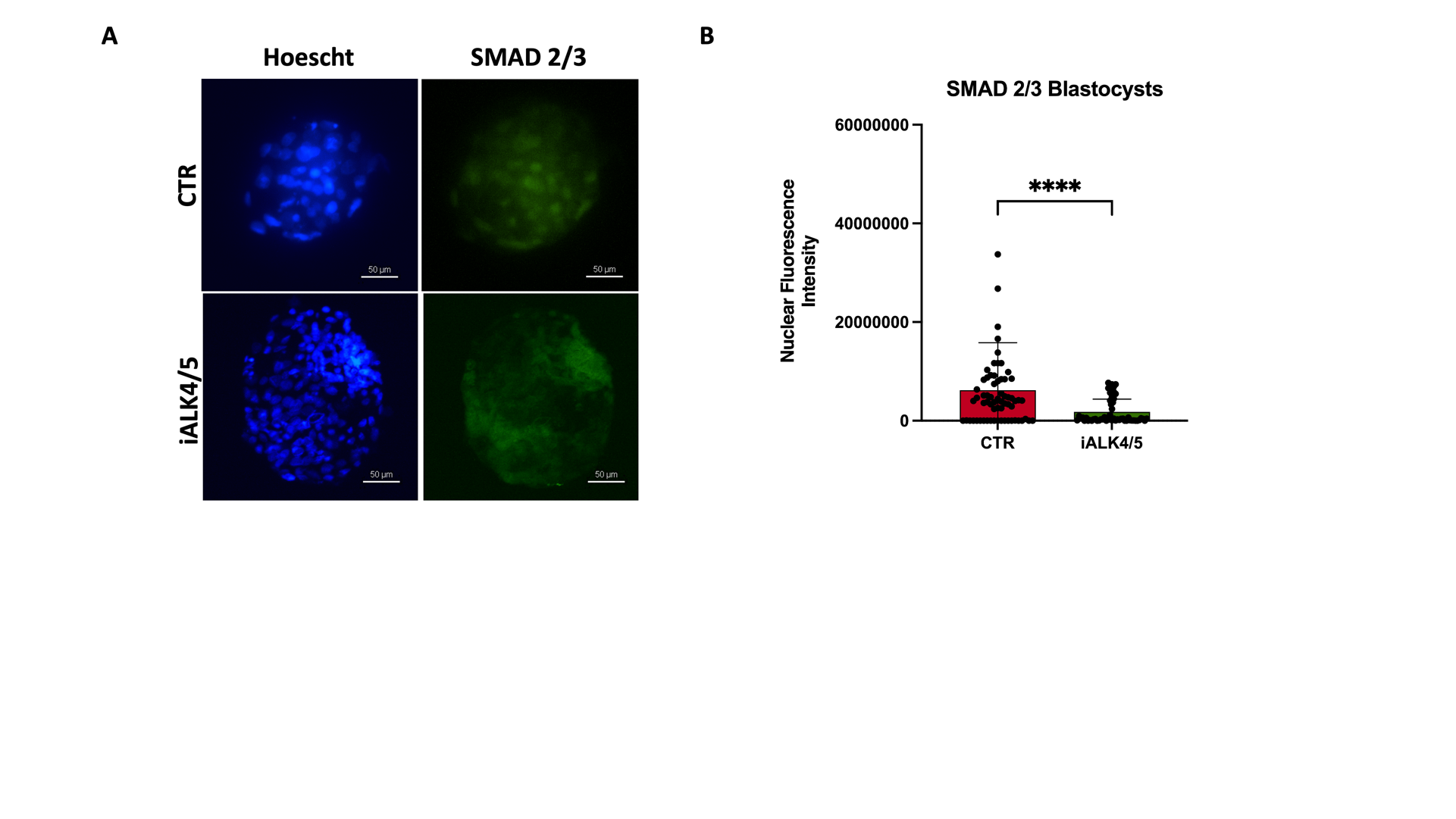
Supplementary Figure 1**

**Supplementary Figure 1. *SM16 effectively inhibits SMAD2/3 nuclear localization in blastocysts.*** (A) Representative fluorescence images of blastocysts cultured under control (CTR) conditions or treated from morula stage with the ALK4/5 inhibitor SM16 (iALK4/5) at 20µM. Immunostaining for SMAD2/3 (green) and nuclear counterstaining with Hoechst (blue) show a marked reduction in nuclear SMAD2/3 signal following SM16 treatment. Images were acquired using a Nikon Eclipse Ti2 fluorescence microscope. Scale bar: 50 µm. (B) Quantification of nuclear SMAD2/3 fluorescence intensity in CTR and SM16-treated blastocysts. Bars represent mean ± SEM; **** P< 0.0001, unpaired two-tailed t-test.
