# Supplementary material for: TGF-β3 promotes trophoblast development in sheep embryos via ACSS2-dependent permissive lipid metabolism
Source: Biol Reprod. 2025 Oct 1;114(3):773–83. doi: 10.1093/biolre/ioaf220 (PMC13017937; doi:10.1093/biolre/ioaf220)
Supplement: Supplementary_Table_ioaf220 [file supplementary_table_ioaf220.docx]

**Supplementary Table 1.** Details of antibodies used for immunostaining

|  | **Target** | **Host Species** | **Source** | **Catalog number** | **Diluition** |  |
| --- | --- | --- | --- | --- | --- | --- |
| Primary Antobodies | OCT4 | Mouse monoclonal | Santa Cruz | sc-365509 | 1:250 | Used in Fig. 2A |
|  | SOX2 | Rat monoclononal | eBioscience | 14-9811-82 | 1:750 | Used in Fig. 5C |
|  | CDX2 | Mouse monoclonal | BioGenex | MU392-UCE | 1:50 | Used in Fig. 2A, 5C |
|  | Phospho- SMAD 2/3 | Rabbit monoclonal | CST | 18338 | 1:50 | Used in Fig. 6A |
|  | SMAD 2/3 | Mouse monoclonal | BD bioscience | 610842 | 1:100 | Used in Fig.6C |
|  | ACSS2 (AceCS1) | Rabbit monoclonal | CST | 3658T | 1:100 | Used in Fig.6E |
| Secondary Antobodies | Anti-Mouse IgG 488 | Goat | Invitrogen | A11001 | 1:750 | Used in Fig. 2A |
|  | Anti-Rat IgG  488 | Goat | Invitrogen | A11006 | 1:750 | Used in Fig. 5C |
|  | Anti-Mouse IgG 594 | Goat | Life Technologies Corp. | A21424 | 1:750 | Used in Fig. 2A, 5C |
|  | Anti-Rabbit IgG  647 | Goat | Life Technologies Corp. | A-21245 | 1:750 | Used in Fig. 6A,E |
